# ANDALE Pittsburgh: results of a *promotora*-led, home-based intervention to promote a healthy weight in Latino preschool children

**DOI:** 10.1186/s12889-018-5266-3

**Published:** 2018-03-16

**Authors:** Sharon E. Taverno Ross, Bethany Barone Gibbs, Patricia I. Documet, Russell R. Pate

**Affiliations:** 1 Department of Health and Physical Activity, University of Pittsburgh, 32 Oak Hill Court, Pittsburgh, PA 15261 USA; 2 Department of Behavioral and Community Health Sciences, University of Pittsburgh, 130 De Soto Street, Pittsburgh, PA 15261 USA; 3 Department of Exercise Science, University of South Carolina, 921 Assembly Street, Columbia, SC 29208 USA

**Keywords:** Hispanic/Latino, Childhood obesity, Preschool, Community health worker, Intervention

## Abstract

**Background:**

Latino preschool children have higher rates of obesity than preschool children from other racial/ethnic groups; however, few effective, culturally appropriate interventions exist targeting this group. The purpose of this study was to test the feasibility of a 10-week, promotora-mediated, home-based intervention to promote a healthy weight in Latino preschool children.

**Methods:**

Trained promotoras (community health workers) delivered 10, 90-min weekly interactive and tailored sessions to Latino families living in Allegheny County. Participants were recruited through promotoras’ own social networks and community gatherings, flyers, and word of mouth. Primary outcome measures included child body mass index (BMI) z-score and percentile. Secondary outcome measures included child objectively measured physical activity and dietary intake, and the home social and physical environment (e.g., parent health behaviors, parent self-efficacy, parental support, physical activity equipment in the home). The final analysis sample included 49 of 51 participants who completed both baseline and follow-up assessments.

**Results:**

Participants included mothers (33.5 ± 6.1 years old) and their preschool-aged children who were primarily 1st generation immigrants from Mexico (65%). The primary analyses of BMI percentile and z-score showed no change post-intervention. However, there was a significant decrease in child BMI percentile for overweight and obese children from baseline to follow-up (*p* < .05). We also saw significant pre/post increases in child daily fruit and vegetable intake, and parent moderate-to-vigorous physical activity, fruit and vegetable servings per day, and self-efficacy; and significant decreases in child saturated fat and added-sugar intake, and child and parent screen time (p’s < .05).

**Conclusions:**

Despite the short duration of the intervention and follow-up, this pilot study showed promising effects of a *promotora*-mediated intervention to promote a healthy weight in Latino preschool children.

**Electronic supplementary material:**

The online version of this article (10.1186/s12889-018-5266-3) contains supplementary material, which is available to authorized users.

## Background

Children of immigrants are the fastest growing segment of the U.S. child population, the majority of which are of Asian or Latino origin [1, 2]. Approximately 16.7% of Latino preschool children are considered obese compared with 3.5% of non-Latino white, 11.3% non-Latino black, and 3.4% of non-Latino Asian children [3]. This is troubling considering obesity tracks into adulthood and puts children and adults at risk for a host of other comorbidities [4–6].

As the U.S. Latino population continues to increase, the public health need for effective, culturally-appropriate obesity interventions for Latino children escalates.

To date, few effective healthy lifestyle interventions exist that target Latino preschool children [7, 8]. There is a particular need for interventions that are inclusive of the entire family unit and include a culturally-sensitive approach. *Promotoras* (i.e., peer health educators who are trusted individuals from the community and share common characteristics with the priority population) [9–11] have been effective in increasing knowledge and promoting behavior changes in Latino populations [12]. *Promotoras* can serve as role models and provide social support to families, empowering them to identify their own needs and implement their own solutions [13]. To our knowledge, only two previous *promotora*-mediated healthy lifestyle interventions have targeted young Latino children and their parents [14, 15]; while the interventions were effective in changing child physical activity and dietary behaviors, they were not effective in reducing child weight status.

As such, the purpose of this pilot study was to test the feasibility of a 10-week, *promotora*-mediated, home-based intervention to promote a healthy weight in Latino preschool children. Using socioecological [16] and Social Cognitive Theory (SCT) [17] as a guide, we targeted changes in the home social (e.g., parental support and self-efficacy) and physical environment (e.g., physical activity equipment, TV in bedroom) associated with improvements in child physical activity, nutrition, and child weight status.

## Methods

### Design

This pilot study followed a single-group pre/post intervention design.

### Participants

ANDALE Pittsburgh (2015–2016) included Latino parents and their 2–5 year old children living in Allegheny county. ANDALE Pittsburgh stands for *Actividad, Nutrición, y Diversión, Apoyando a los Latinos En Pittsburgh* (translated as Physical activity, nutrition, and Fun, Supporting Latinos in Pittsburgh). Allegheny county can be described as an emerging Latino community, i.e., anarea with low (< 5%) yet growing concentrations of Latinos [18]. Latinos living in this community are scattered throughout the region with no concentration in a single neighborhood or area [19, 20], and face barriers to health care, legal, and social services [19, 21]. *Promotoras* recruited participants through their own social networks (e.g., schools, churches, neighborhood) as well as at community gatherings, flyers, and word of mouth. Study staff screened interested participants for eligibility on the phone or in-person. Eligibility included that the participating parent: (1) self-identifies as Hispanic/Latino, (2) has at least one child between 2 and 5 years old, and (3) speaks Spanish or English. Participants were ineligible if the participating child had a condition that would invalidate the measure of physical activity (e.g., wheelchair-bound), or the parent primarily spoke a language other than Spanish or English.

### *Promotoras* recruitment/training

Nine *promotoras* (females > 18 years, active in community, ability to read/write in Spanish) were recruited through several community-based organizations, preexisting community contacts, and word of mouth. *Promotoras* received 25 h of training delivered by the Project Coordinator using a train-the-trainer model and the intervention curriculum finalized after a year-long developmental phase (described below). Specifically, training topics included *promotora* core roles, and skills-, health-, and research-based knowledge, as well as orientation to and role play with the intervention curriculum. Additional details of the intervention development and intervention description for ANDALE Pittsburgh have been published previously and can be found elsewhere [22].

### Intervention description

This study was guided by a socioecological framework [16] and the SCT [17]. It was developed over a year-long process of conducting formative research with parents and key stakeholders in the community, as well as input from a community coalition and two research advisory boards. The intervention focused on improving dietary intake, decreasing sedentary behavior, and increasing physical activity using the 5,2,1,0 message (5 or more servings of fruits and vegetables, 2 h or less of recreational screen time, 1 h or more of physical activity, and 0 sugary drinks and more water) [23]. *Promotoras* delivered the home-based, face-to-face intervention to families over 10, 90-min weekly sessions that included education (i.e., session content related to the topic), practice (i.e., hands-on activities and role play), and action (i.e., goal setting and problem solving). Select intervention topics included a healthy lifestyle (i.e., diet and physical activity), reducing sedentary time, healthy eating for the entire family, and community nutrition and physical activity resources. Behavior modification constructs and strategies (e.g., goal setting, problem solving, social support), along with building of self-efficacy through healthy recipe preparation and physical activity breaks, were included. Additional details of the intervention development and intervention description can be found elsewhere [22].

### Data collection

Data on child physical activity were collected via accelerometry; measures of child diet and the home social and physical environment were assessed via parent survey. A trained bilingual data collector visited participating families’ homes with the *promotora* to get informed consent and deliver the accelerometer for the child to wear 7–10 days before Session #1. Parents received detailed verbal and written instructions on how and when children should wear the accelerometers. The data collector completed the survey and anthropometric measures during the first and last home visits (#1 and #10). At Session #10, the data collector also distributed the accelerometers for the children to wear during the following 7 days, and picked them up upon completion of the study. The Institutional Review Board at the University of Pittsburgh approved all study protocols.

### Outcomes

For the current study, the primary outcomes included child BMI z-score and percentile. Secondary outcomes included child physical activity and diet, and the home social and physical environment (e.g., parent health behaviors, parent self-efficacy, parental support, physical activity equipment in the home).

### Measures

#### Anthropometry and weight status

Parent and child height and weight were measured in light clothing and without shoes using a Seca model 213 mobile stadiometer and Seca model 869 digital scale. Body mass index (BMI) was calculated using the standard equation (body weight [kg] / height [m]^2^), and children were classified as normal weight (BMI percentile < 85) or overweigh/obese (BMI percentile ≥85) based on Centers for Disease Control (CDC) growth charts [24].

#### Physical activity

Child physical activity was measured by ActiGraph GT3X (Pensacola, FL) accelerometers during a 7-day period. Children wore the monitors on an elastic belt on their right hip. Parents received instructions to remove the monitor only during sleep or water-related activities (e.g., bathing, swimming). Data was collected and stored in 15-s intervals to capture the sporadic activity patterns that are typical of young children. Data were reduced using ActiLife version 6 software with nonwear time defined as 60 min of 0 counts and activity intensity cut-points developed for preschool-aged children [25, 26]. Accelerometry data was considered valid if participants had ≥8 h of wear time on ≥3 days at both baseline and follow-up (*n* = 22) [27]. For each participant, physical activity (min/h) and counts (per min) were averaged over accelerometer wear time.

Parent physical activity was measured via self-report using three items adapted from a validated survey [28] and translated into Spanish. To estimate screen time, parents were also asked, on an average day, how many hours they spent watching TV, DVDs, or videos. Parents also reported how much time their child spent watching TV, playing or working on the internet/computer, and playing video games, per day.

#### Diet

Child dietary intake was assessed via English or Spanish-language versions of the validated Block Food Screener for Kids 2007 (NutritionQuest, Berkeley, CA) [29]. Parents completed this 15–20 min screener to assess children’s dietary intake from the past week. A version of this screener has been used previously with Latino children [30]. Data were processed to estimate child’s intake of whole/processed fruit (includes apples, applesauce, and other fruit; grams per day), vegetables (without potatoes; cups per day), whole grains (ounces per day), saturated fat (grams per day), added sugar and syrup (teaspoons per day), and sugar-sweetened beverages (calories per day).

#### Home environment

To assess the social and physical home environment related to diet and physical activity, parents completed a survey adapted from several sources and translated into Spanish. Physical activity items included physical activity resources and media availability [31], parent physical activity self-efficacy [32], modeling behaviors [33], and support for child physical activity [34]. Diet items included meal and feeding behaviors [35, 36] and support for child healthy eating [37]. Parenting strategies related to children’s diet and physical activity were also assessed [38].

#### Demographics

Demographic variables were assessed via parent report at baseline and included information on parent and child age and gender, country of origin, parent marital status, employment status, highest education in household, and household income. Acculturation was measured using the Brief Acculturation Scale for Hispanics; the average of four questions about preferred language in different contexts was calculated and ranged from 0 (only Spanish, low acculturation) to 1 (only English, high acculturation) [39].

### Sample size calculations

Sample size and power calculations for the intervention were based on repeated measures ANOVA with pre- to post-intervention change in BMI z-score as the primary outcome. We used GPower 3 software for all calculations. Based on data from a previous child obesity intervention with 2–4-year-old Latino children (mean decrease in BMI z-score of .20, SE = 0.80) [40], we expected that, on average, children participating in the intervention will slightly decrease their BMI z-score. We anticipated an effect size of .15 to .20, with correlations between .60 to .80. With 50 parent-child dyads, we had 65% to 90% power using a two-sided t-test and 5% significance level.

### Statistical analyses

All data analyses were performed using Stata version 14 (College Station, TX). Descriptive statistics for baseline sociodemographic characteristics are summarized as either means and standard deviations or percentages and sample size. Changes from pre- to post-intervention were tested for statistical significant using paired *t* tests or nonparametric Wilcoxon signed-rank tests for categorical, ordinal, or non-normally distributed data. Changes in child anthropometric measures were repeated after stratification by BMI percentile (<85th, ≥85th). Statistical significance was set at *p* < 0.05.

## Results

Of 51 parent-child dyads enrolled in the home-based intervention ANDALE Pittsburgh (2015–2016), 49 (96%) completed the intervention and assessments, and were included in analyses. Table 1 reports sociodemographic characteristics of the participants. The majority of the children were male (59.2%; average age 3.9 ± 1.3), and all of the parents who completed baseline and follow-up assessments were mothers (100%; average age 33.5 ± 6.1 years). The majority of mothers were from Mexico (65%) with low acculturation (0.14 ± 0.17), and 67.3% of the sample had lived in the U.S. for 6 or more years; 98% of the mothers were married and 71% reported being stay-at-home caregivers. The highest household education level was almost equally split between high school or less (48%) or more than high school (52%). Forty-seven percent of families reported an annual household income of $34,999 or less, 20% reported above $35,000, and 33% didn’t know/refused. Approximately 53% of children were categorized as normal weight, 18% were categorized as overweight, and 29% were categorized as obese.

**Table 1:** Sociodemographic characteristics (% [n], or M ± SD) of participants (*n* = 49) in the ANDALE Pittsburgh home-based intervention

Characteristic	
Child gender, % female	40.8% (20)
Child Age, years	3.9 ± 1.3
Parent gender, % female	100%, (49)
Parent Age	33.5 ± 6.1
Country of Origin	
Mexican	32 (65%)
Guatemalan	4 (8%)
Columbian	4 (8%)
Venezuelan	4 (8%)
Other	5 (10%)
Marital Status	
Married or in a committed relationship	48 (98%)
Divorced/separated	1 (2%)
Employment (parent)	
Working full time	4 (8%)
Working part time	8 (16%)
Stay at home caregiver	35 (71%)
Currently unemployed, but seeking work	2 (4%)
Education (highest in household)	
Did not finish high school	7 (14%)
Finished high school or GED	16 (33%)
Some college or training after high school	10 (20%)
Finished college	10 (20%)
Advanced degree	6 (12%)
Income	
Less than $20,000	17 (35%)
$20,000–34,999	6 (12%)
$35,000–49,999	4 (8%)
$50,000–74,999	2 (4%)
$75,000–99,999	1 (2%)
$100,00 or more	3 (6%)
Don’t know/refused	16 (33%)
Acculturation	
Low (0–0.25)	42 (86%)
Moderate (0.26–0.50)	5 (10%)
High (0.51–1)	2 (4%)

Table 2 includes anthropometric variables at baseline, follow-up and change scores for children, overall and among children that had normal (<85th percentile, *n* = 26) or overweight/obese (≥85th percentile, *n* = 23) BMI. Overall or within strata, child weight (kg) increased significantly from baseline to follow-up. There were no other significant changes in child BMI z-score, BMI percentile, or waist circumference in the total sample. Noteworthy, however, was a significant decrease in BMI percentile among children who were overweight at the beginning of the intervention (− 1%, *p* = 0.013). Also, there was a significant change in the distribution of normal vs. overweight/obese across follow-up (Fisher’s exact test, *p* < 0.001), with 1 of 26 normal weight children transitioning to overweight/obese and 5 of 23 overweight/obese children transitioning to normal weight.

**Table 2:** Anthropometric measures at baseline, follow-up, and change scores (mean ± SD or median [25th, 75th percentile]), among children participating in ANDALE Pittsburgh by weight status [overall (*n* = 49), and in children with body mass index percentile <85th (*n* = 26) or ≥ 85th (*n* = 23)]

		Baseline	Follow-up	Change Score	*p*-value
Child weight, kg	Overall	18.5 ± 5.3	19.0 ± 5.3	0.5 ± 0.8	**0.002**
	<85th percentile	16.7 ± 3.1	17.1 ± 3.2	0.4 ± 0.6	**0.003**
	≥85th percentile	20.6 ± 6.5	21.1 ± 6.4	0.5 ± 1.0	**0.013**
Child BMI z-score ^a^	Overall	0.96 ± 1.29	0.91 ± 1.35	−0.05 ± 0.44	0.437
	<85th percentile	0.08 ± 0.06	0.02 ± 0.08	−0.05 ± 0.04	0.535
	≥85th percentile	2.00 ± 1.06	1.96 ± 1.11	− 0.05 ± 0.47	0.643
Child BMI percentile ^a^	Overall	83 [43, 96]	82 [50, 94]	0 [−7, 2]	0.430
	<85th percentile	45 [31, 74]	53 [26, 77]	1.5 [−10, 10]	0.557
	≥85th percentile	96 [90, 99]	94 [87, 99]	−1 [−3, 0]	**0.013**
Child waist circumference, cm	Overall	56.0 ± 10.5	55.7 ± 7.5	−0.3 ± 7.4	0.797
	<85th percentile	52.7 ± 10.3	52.6 ± 4.2	0.1 ± 9.9	0.969
	≥85th percentile	59.6 ± 9.7	59.2 ± 8.9	−0.5 ± 2.7	0.398

NOTE: Data were compared using paired *t* tests or nonparametric Wilcoxon signed-rank tests; Baseline vs. follow-up data compared using paired *t* test or a nonparametric sign test;

*BMI* body mass index;

^a^One child’s BMI was too high to calculate a z-score or percentile at baseline or follow-up;

*p*-values <0.05 were bolded to indicate statistical significance

Table 3 includes child dietary intake, physical activity, and screen time variables at baseline, follow-up, and change scores. There was a significant increase in daily intake of both fruit (+ 4.43 ± 1.26 g per day, *p* = 0.001) and vegetables (+ 0.14 ± 0.06 cups per day, *p* = 0.034). Children also had significant decreases in saturated fat (− 3.0 ± 0.6 g per day, *p* < 0.001), added sugar/syrup (− 1.63 ± 3.03 teaspoons per day, p < 0.001), and calories from sugar-sweetened beverages (− 7.8 ± 26.0 kcals per day, *p* = 0.040). Of children meeting minimum wear-time requirements for the objective activity assessment, average wear time was 12.2 ± 1.6 h on 5.0 ± 1.2 days. There were no statistically significant changes from baseline to follow-up in any of the physical activity variables (accelerometry) or average counts per minute. However, there was a statistically significant decrease in parent-reported minutes per day of child screen time (60 [35, 120] vs. 60 [32, 90]; *p* = 0.02).

**Table 3:** Diet, physical activity, and screen time at baseline, follow-up, and change scores (M ± SD) among children participating in ANDALE Pittsburgh intervention

	Baseline	Follow-up	Change Score	*p*-value
*Dietary Intake (n = 49)*				
Fruit, grams per day	19.29 ± 1.27	23.71 ± 1.39	4.43 ± 1.26	**0.001**
Vegetables (no potatoes), cups per day	0.49 ± 0.42	0.63 ± 0.60	0.14 ± 0.06	**0.034**
Whole Grains, oz. per day	0.72 ± 0.64	0.74 ± 0.87	0.02 ± 0.65	0.790
Saturated Fat, grams per day	15.2 ± 7.3	12.2 ± 7.4	− 3.0 ± 0.6	**< 0.001**
Sugar/syrup added to foods/beverages, tsp per day	5.06 ± 3.58	3.43 ± 2.21	− 1.63 ± 3.03	**< 0.001**
Sugar-Sweetened Beverages, kcals per day	15.5 ± 26	7.6 ± 12.1	−7.8 ± 26.0	**0.040**
*Objectively-measured Physical Activity (n = 22)*				
Sedentary behavior, min/h	34.7 ± 3.4	34.8 ± 4.3	0.1 ± 0.8	0.942
Very Light, min/h	12.3 ± 1.7	12.2 ± 2.0	− 0.2 ± 1.2	0.531
Light Activity, min/h	6.5 ± 1.1	6.8 ± 1.3	0.2 ± 1.0	0.308
Moderate Activity, min/h	4.7 ± 1.3	4.8 ± 1.6	0.1 ± 0.3	0.770
Vigorous Activity, min/h	1.7 ± 1.1	1.4 ± 1.1	− 0.2 ± 0.8	0.223
Total Activity, min/h	25.2 ± 3.4	25.2 ± 3.4	−0.1 ± 3.5	0.942
Average Counts per Minute	543 ± 145	532 ± 35	−12 ± 133	0.681
*Screen Time (n = 49)*				
Screen time, minutes/day	60 [35, 120]	60 [32, 90]	0 [−60, 0]	**0.020**

Data are reported as mean ± SD or n (%) across ordinal categories. Data were compared using paired *t* tests or nonparametric Wilcoxon signed-rank tests

*p*-values <0.05 were bolded to indicate statistical significance

**Fig. 1:**
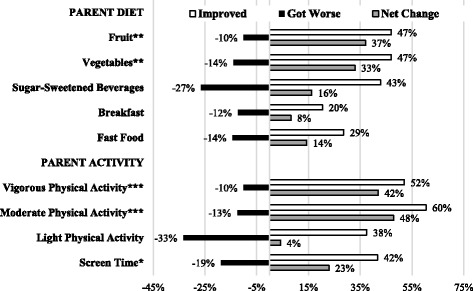
Parent diet, physical activity, and screen time outcomes in the ANDALE Pittsburgh intervention (*n* = 49). Parents answered semi-quantitative questions at baseline and follow-up. Relative frequency of improving (white bar), worsening (black bar), and the net change (gray bar) are presented. *p < 0.05; **p < 0.01; ***p < 0.001 for significant improvement over time by the Wilcoxon test

Figure 1 and Additional file 1: Table S1 include dietary intake, physical activity, screen time, and anthropometric variables for parents at baseline, follow-up, and change scores from semi-quantitative questionnaires. In Fig. 1, the proportion of parents improving, worsening, and the net improvement in dietary and activity behaviors are displayed. Parents significantly improved (net change indicated by gray bars) intake of fruits (*p* = 0.001) and vegetables (*p* = 0.002), engagement in vigorous physical activity (*p* < 0.001) and moderate physical activity (*p* < 0.001), and screen time (*p* = 0.016). No changes were observed in anthropometric measures (Additional file 1: Table S1).

Additional file 2: Table S2 includes features of the home social and physical environment at baseline, follow-up, and change scores, for parent-child dyads participating in ANDALE Pittsburgh (*n* = 49). In general, we did not detect statistically significant differences in measures of the social and physical home environment to promote physical activity and nutrition in the children. However, parent reported self-efficacy to overcome barriers to physical activity significantly increased from baseline to follow-up (*p* = 0.024).

## Discussion

ANDALE Pittsburgh is one of the first healthy lifestyle interventions that was delivered by *promotoras* and targeted Latino preschool-aged children and their families. Despite the short duration of intervention and follow up, we saw significant pre/post improvements in both child and parent self-reported dietary intake and screen time, and parent physical activity. These findings suggest that a *promotora-*mediated intervention with Latino preschool children living in an emerging Latino community may be effective in improving both child and parent behaviors associated with excessive weight gain (i.e., physical activity, dietary intake, sedentary behavior).

Previous healthy lifestyle programs with *promotoras* targeting young Latino children have had some success in improving physical activity and nutrition. For example, one previous 3-year intervention, Aventuras Para Niños study, targeted young Latino children (K-2nd grade) and their parents [14]. In the school/community-level arm of the intervention of this study, parents reported increased child physical activity, reduced child frequency of watching TV when getting ready for school, and increased child’s daily consumption of fruits and vegetables. Conversely, similar results were not observed in the intervention arm delivered in homes by *promotoras*. In another 9-month intervention study by Bender et al. [15] targeting low-income Mexican American mothers and their 3–5 year old children, the authors found significant decreases in child sugar-sweetened beverage consumption, and significant increases in child water consumption and maternal step counts measured by pedometers. However, in both of these studies, they did not see expected improvements in child weight status.

We saw a significant reduction in BMI percentile for those children who were overweight or obese at baseline. These results are similar to previous studies with young Latino children where the strongest intervention effect was seen in the obese children [40–42], while others saw no effect [14, 15, 43, 44]. In general, results have been mixed regarding intervention effects on BMI of preschool-aged children [7, 8, 45, 46]. While we would not necessarily expect a decrease in BMI for those growing and normal weight preschool children, it is unclear whether the lack of change over the intervention period (i.e., maintenance of BMI z-score or percentile) can be defined as success without the comparison of a control group. For example, it’s possible we would have seen an increase in BMI z-score or percentile over the intervention period in the control group. As such, the intervention should be evaluated on a larger scale, with a longer intervention and follow-up period, using an experimental design with a control group, as well as adequate sample sizes across the BMI categories (normal, overweight, obese).

A recent decade review of cross-sectional and longitudinal studies examining home environmental influences on obesity in Latino children found that key factors included: a) parental influences (e.g., feeding practices, modeling), b) screen time behaviors and rules, c) child and parent physical activity/sedentary behavior, d) socioeconomic status/food security, and e) sleep deprivation [47]. According to our theoretical framework, we hypothesized that changes in child behaviors and weight status would occur through changes in the home social and physical environment and modeling of healthy parent behaviors. While we saw significant improvements in parent physical activity and nutrition behaviors, the only significant change related to the home social and physical environment was parent self-efficacy to overcome physical activity barriers. Previous studies have had some success in improving Latino children’s home social and physical environment related to physical activity and nutrition [14, 48, 49]. However, much remains to be understood about the most important early risk and protective factors for Latino child obesity, and how best to tailor intervention approaches to Latino families and the home-environment.

There is little known about Latinos living in these emerging Latino communities, even less about determinants of obesity and potentially effective intervention approaches. A major strength of this study is that it was one of the first to examine the feasibility of a *promotora-*led, healthy lifestyle intervention in Latino preschool children living in an emerging community. Further, our sample of parents and children was representative of the racial/ethnic and socioeconomic background of the Latino population living in this community. Another strength is the excellent retention of the sample across the 10-week intervention. However, the study is not without limitations. Given the small sample size of the pilot study, we were not adequately powered to examine differences in these results by gender, BMI, or physical activity level. Further, while child physical activity was measured objectively by accelerometry, the criteria for adequate wear time limited number of children with complete data to only *n* = 22, which may have prevented us from seeing any meaningful pre/post differences.

## Conclusions

In conclusion, there are clear disparities in child obesity for preschool-aged Latino children, and there is a lack of knowledge surrounding effective intervention approaches to promote a healthy weight this population. The present study suggests a *promotora* intervention is associated with self-reported changes in important behaviors linked to excessive weight gain. The results of this pilot study are promising and suggest the need to examine the intervention in a longer and larger, experimental study with a control group to confirm and extend our findings. If successful, this research could provide a potential model to help to address and prevent obesity and promote a healthy weight in Latino families with preschool children, a highly significant and growing public health problem.

## Additional files

Additional file 1: **Table S1.** Parent weight, diet, physical activity, and screen time outcomes in the ANDALE Pittsburgh intervention (*n* = 49). Pre/post and change scores for parent-level outcome variables assessed in the intervention. (DOCX 16 kb)

Additional file 2: **Table S2.** Home environment at baseline and follow-up, and change scores, in the ANDALE Pittsburgh intervention (*n* = 49). Pre/post and change scores for home-level outcome variables assessed in the intervention. (DOCX 19 kb)

## Abbreviations

BMI: Body mass index
CDC: Centers for disease control
SCT: Social cognitive theory
