# Supplementary material for: ANDALE Pittsburgh: results of a promotora-led, home-based intervention to promote a healthy weight in Latino preschool children
Source: BMC Public Health. 2018 Mar 16;18:360. doi: 10.1186/s12889-018-5266-3 (PMC5857096; doi:10.1186/s12889-018-5266-3)
Supplement: Supplementary file 1 — Table S1. Parent weight, diet, physical activity, and screen time outcomes in the ANDALE Pittsburgh intervention (n = 49). Pre/post and change scores for parent-level outcome variables assessed in the intervention. (DOCX 16 kb) [file 12889_2018_5266_MOESM1_ESM.docx]

**Additional file Table 1. Parent weight, diet, physical activity, and screen time outcomes in the ANDALE Pittsburgh intervention (n=49)**

|  | Pre | Post | Change | p-value |
| --- | --- | --- | --- | --- |
| **Weight and adiposity** |  |  |  |  |
| Parent weight, kg | 71.4 ± 19.3 | 71.3 ± 19.1 | -0.1 ± 1.6 | 0.615 |
| Parent BMI, kg/m^2^ | 29.0 ± 7.3 | 28.9 ± 7.2 | -0.1 ± 0.1 | 0.465 |
| Parent waist, cm ^b^ | 96.1 ± 15.8 | 95.5 ± 14.2 | -0.6 ± 7.0 | 0.557 |
| Parent waist-to-hip ratio ^b^ | 0.919 ± 0.099 | 0.915 ± 0.085 | -0.004 ± 0.070 | 0.718 |
| **Diet** |  |  |  |  |
| Parent Fruit, servings per day  0  <1  1-2  3-4  5 or more | 2 (4%)  8 (16%)  28 (57%)  5 (10%)  6 (12%) | 0  1 (2%)  24 (49%)  17 (35%)  7 (14%) | 23 increased; 5 decreased | **0.001** |
| Parent Vegetables, servings per day  0  <1  1-2  3-4  5 or more | 3 (6%)  14 (29%)  27 (55%)  5 (10%)  0 | 1 (2%)  6 (12%)  30 (61%)  8 (16%)  4 (8%) | 23 increased  7 decreased | **0.002** |
| Parent Sugar-Sweetened Beverages, drinks per week  <1 drinks per week  1 drinks per week  2-4 drinks per week  5-6 drinks per week  1 per day  2 or more per day | 18 (37%)  10 (20%)  10 (20%)  2 (4%)  6 (12%)  3 (6%) | 19 (39%)  19 (39%)  7 (14%)  1 (2%)  2 (4%)  1(2%) | 13 increased;  21 decreased | 0.062 |
| Parent Breakfast, days per week**^a^**  Never  1-2 days  3-4 days  5-6 days  7 days | 4 (8%)  6 (13%)  4 (8%)  4 (8%)  30 (63%) | 1 (2%)  4 (8%)  6 (13%)  6 (13%)  31 (65%) | 10 increased; 6 decreased | 0.256 |
| Parents Fast food, times per week  Never  1-2  3-4  5-6  7  More than 7 | 15 (31%)  34 (69%)  0  0  0  0 | 23 (47%)  25 (51%)  1 (2%)  0  0  0 | 7 increased; 14 decreased | 0.127 |
| **Physical Activity** |  |  |  |  |
| Parent vigorous PA  None  <½ hours a week  ½ - 2 hours a week  2½ - 4 hours a week  4½ - 6 hours a week  6 or more hours a week | 26 (54%)  3 (6%)  13 (27%)  4 (8%)  2 (2%)  2 (2%) | 13 (27%)  6 (13%)  15 (31%)  10 (21%)  2 (4%)  2 (4%) | 25 increased; 5 decreased | **<0.001** |
| Parent moderate PA  None  <½ hours a week  ½ - 2 hours a week  2½ - 4 hours a week  4½ - 6 hours a week  6 or more hours a week | 15 (31%)  7 (15%)  16 (33%)  7 (15%)  1 (2%)  2 (4%) | 3 (6%)  9 (19%)  16 (33%)  4 (8%)  10 (21%)  6 (13%) | 29 increased;  6 decreased | **<0.001** |
| Parent light PA  None  <½ hours a week  ½ - 2 hours a week  2½ - 4 hours a week  4½ - 6 hours a week  6 or more hours a week | 1 (2%)  2 (4%)  8 (17%)  8 (17%)  6 (13%)  23 (48%) | 0  6 (13%)  5 (10%)  8 (17%)  6 (13%)  23 (48%) | 18 increased; 16 decreased | 0.788 |
| Parent screen time hrs/day | 2 [1, 2] | 1 [½ , 2] | 9 increased; 20 decreased | **0.016** |

Data are reported as mean ± SD or n (%) across ordinal categories. Data were compared using paired *t* tests or nonparametric Wilcoxon signed-rank tests.

^a^ missing n=2

^b^ missing n=1
