# Supplementary material for: ANDALE Pittsburgh: results of a promotora-led, home-based intervention to promote a healthy weight in Latino preschool children
Source: BMC Public Health. 2018 Mar 16;18:360. doi: 10.1186/s12889-018-5266-3 (PMC5857096; doi:10.1186/s12889-018-5266-3)
Supplement: Supplementary file 2 — Table S2. Home environment at baseline and follow-up, and change scores, in the ANDALE Pittsburgh intervention (n = 49). Pre/post and change scores for home-level outcome variables assessed in the intervention. (DOCX 19 kb) [file 12889_2018_5266_MOESM2_ESM.docx]

**Additional file Table 2 – Home environment at baseline and follow-up, and change scores, among parents in the ANDALE Pittsburgh intervention (n=49)**

| **Social Environment** | Pre | Post | Change | p-value |
| --- | --- | --- | --- | --- |
| **Family meals** |  |  |  |  |
| Family Meal, times per week  Never  1-2  3-4  5-6  7  More than 7 | 0  2 (9%)  3 (6%)  12 (24%)  12 (24%)  16 (33%) | 0  9 (18%)  7 (14%)  9 (18%)  12 (25%)  12 (25%) | 10 increased; 18 decreased | 0.152 |
| Family Fast Food, times per week  Never  1-2  3-4  5-6  7  More than 7 | 17 (35%)  31 (63%)  1 (2%)  0  0  0 | 19 (39%)  30 (61%)  0  0  0  0 | 6 increased; 9 decreased | 0.439 |
| **Intangible support** |  |  |  |  |
| Praise child for PA  Never  Rarely  Sometimes  Often  Always | 0  0  8 (17%)  9 (19%)  31 (65%) | 0  1 (2%)  8 (17%)  10 (21%)  29 (60%) | 8 increased; 10 decreased | 0.614 |
| Praise child for healthy snack^a^  Never  Rarely  Sometimes  Often  Always | 11 (24%)  6 (13%)  5 (11%)  8 (17%)  16 (34%) | 11 (24%)  1 (2%)  11 (24%)  9 (20%)  14 (30%) | 16 increased; 12 decreased | 0.579 |
| PA Encouragement, number of days  0 per week  1-2 per week  3-4 per week  5-6 per week  7 per week | 4 (8%)  18 (38%)  11 (23%)  7 (15%)  8 (17%) | 3 (6%)  9 (19%)  19 (40%)  9 (19%)  8 (17%) | 21 increased; 9 decreased | 0.102 |
| **Tangible Support** |  |  |  |  |
| Modeling^b^  0 per week  1-2 per week  3-4 per week  5-6 per week  7 per week | 8 (17%)  19 (40%)  11 (23%)  4 (9%)  5 (11%) | 3 (6%)  17 (36%)  15 (32%)  11 (23%)  1 (2%) | 22 increased, 11 decreased | 0.090 |
| Transportation  0 per week  1-2 per week  3-4 per week  5-6 per week  7 per week (daily) | 11 (23%)  22 (46%)  10 (21%)  4 (8%)  1 (2%) | 8 (17%)  21 (43%)  11 (23%)  8 (17%)  0 | 16 increased; 9 decreased | 0.196 |
| Watching  0 per week  1-2 per week  3-4 per week  5-6 per week  7 per week (daily) | 8 (17%)  25 (53%)  6 (13%)  4 (8%)  5 (10%) | 7 (15%)  20 (42%)  11 (23%)  8 (17%)  2 (4%) | 18 increased; 12 decreased | 0.399 |
| **PA Attitudes** |  |  |  |  |
| Self-efficacy for PA (summary on a 5-point scale, 5 being high) | 2.1 ± 0.7 | 2.3 ± 0.7 | 0.3 ± 0.7 | **0.024** |
| Important child engage in sports/PA  Not important  Somewhat unimportant  Somewhat important  Very important | 0  1 (2%)  15 (31%)  32 (67%) | 0  0  18 (38%)  30 (53%) | 8 increased; 7 decreased | 0.796 |
| Important active as family  Not important  Somewhat unimportant  Somewhat important  Very important | 0  1 (2%)  17 (35%)  37 (77%) | 0  0  10 (21%)  37 (77%) | 13 increased; 5 decreased | 0.071 |
| Important family eat together  Not important  Somewhat unimportant  Somewhat important  Very important | 1 (2%)  0  4 (8%)  43 (90%) | 2 (4%)  0  1 (2%)  45 (94%) | 3 increased; 1 decreased | 0.333 |
|  |  |  |  |  |
| **Physical Environment** |  |  |  |  |
| TV in child’s bedroom, n (%) | 22 (46%) | 26 (54%) | 6 increased; 2 decreased | 0.157 |
| PA Equipment at home (sum) | 4.6 ± 2.6 | 5.0 ±2.7 | 0.4 ± 2.6 | 0.236 |

Data are reported as mean ± SD or n (%) across ordinal categories. Data were compared using paired *t* tests or nonparametric Wilcoxon signed-rank tests.

^a^ missing n=2

^b^ missing n=1
